# Triantennary GalNAc-Functionalized Multi-Responsive Mesoporous Silica Nanoparticles for Drug Delivery Targeted at Asialoglycoprotein Receptor

**DOI:** 10.3390/ijms23116243

**Published:** 2022-06-02

**Authors:** Rosemeyre Cordeiro, Ana Carvalho, Luísa Durães, Henrique Faneca

**Affiliations:** 1 CNC—Center for Neuroscience and Cell Biology, Faculty of Medicine, University of Coimbra, Polo I, 3004-517 Coimbra, Portugal; rcordeiro@uc.pt (R.C.); amcarv95@gmail.com (A.C.); 2 Institute for Interdisciplinary Research (IIIUC), University of Coimbra, 3030-789 Coimbra, Portugal; 3 CIEPQPF, Department of Chemical Engineering, University of Coimbra, Rua Sílvio Lima, Polo II, 3030-790 Coimbra, Portugal; luisa@eq.uc.pt

**Keywords:** mesoporous silica nanoparticles, *N*-acetylgalactosamine, epirubicin, targeting

## Abstract

In recent years, mesoporous silica particles have been revealed as promising drug delivery systems combining high drug loading capacity, excellent biocompatibility, and easy and affordable synthetic and post-synthetic procedures. In fact, the straightforward functionalization approaches of these particles allow their conjugation with targeting moieties in order to surpass one of the major challenges in drug administration, the absence of targeting ability of free drugs that reduces their therapeutic efficacy and causes undesired side effects. In this context, the main goal of this work was to develop a new targeted mesoporous silica nanoparticle formulation with the capability to specifically and efficiently deliver an anticancer drug to hepatocellular carcinoma (HCC) cells. To this purpose, and as proof of concept, we developed redox-responsive mesoporous silica nanoparticles functionalized with the targeting ligand triantennary *N*-acetylgalactosamine (GalNAc) cluster, which has high affinity to asialoglycoprotein receptors overexpressed in HCC cells, and loaded them with epirubicin, an anthracycline drug. The produced nanocarrier exhibits suitable physicochemical properties for drug delivery, high drug loading capacity, high biocompatibility, and targeting ability to HCC cells, revealing its biopharmaceutical potential as a targeted drug carrier for therapeutic applications in liver diseases.

## 1. Introduction

Mesoporous silica nanoparticle (MSN)-based systems have aroused great interest in the field of drug delivery due to numerous advantages. In fact, not only their structural properties (large surface area and pore volume) allow for a good loading capacity and easy drug encapsulation; however, their simple, scalable, and cost-effective production are attractive features in the development of a drug delivery system, namely for application in cancer treatment [1,2,3]. Their easy synthetic and post-synthetic procedures allow them to incorporate specific characteristics, such as stimuli-responsive drug release behavior, or functionalization with molecules of interest (such as ligands) to direct them to target cells [2]. The latter is particularly relevant in chemotherapy strategies, since they are strongly associated with severe side effects due to off-target undesired toxicity. It is known that, in general, cancer cells overexpress certain receptors that can be used as targets for selective delivery, mediated by nanocarriers, to these cells. By modifying the surface of the nanosystems with a ligand that interacts specifically with one of these overexpressed receptors, the nanocarriers can directly target the tumor cells, reducing the side effects. One example is the ligand triantennary *N*-acetylgalactosamine (GalNAc), developed by Ionis Pharmaceuticals Inc., that has high affinity for the asialoglycoprotein receptor (ASGP-R), which is overexpressed in hepatocellular carcinoma cells (HCC) [4]. There are several research studies using this ligand to carry nucleic acids to hepatocytes—conjugating it directly with antisense oligonucleotides (ASO) [5,6] or with short interfering RNAs [7,8,9]—and to carry protein, conjugating it with biotin, antibodies, or fragments of antibodies to act as degraders [10]. There is an FDA- and EMA-approved *N*-acetylgalactosamine-conjugated RNA interference therapeutic—GIVLAARI^®^ (Alnylam^®^ Pharmaceuticals), also known by givosiran—to treat acute hepatic porphyria in clinical trials along with 20 other GalNAc-siRNA or GalNAC-ASO conjugates [11]. GalNAc ligand has also been employed in carriers for nucleic acid delivery based on lipids [12,13], polymers [14], or silica-polymers [15] to confer them specificity to hepatic cells. However, this ligand has not been widely explored to develop targeted drug delivery systems. Petrov et al. developed docetaxel-GalNAc conjugates [16], and recently, our group developed a polymer–lipid nanoparticle formulation, functionalized with this ligand to transport and deliver selumetinib and sorafenib in HCC [17], one of the main causes of cancer-related death [18].

**Scheme 1:**
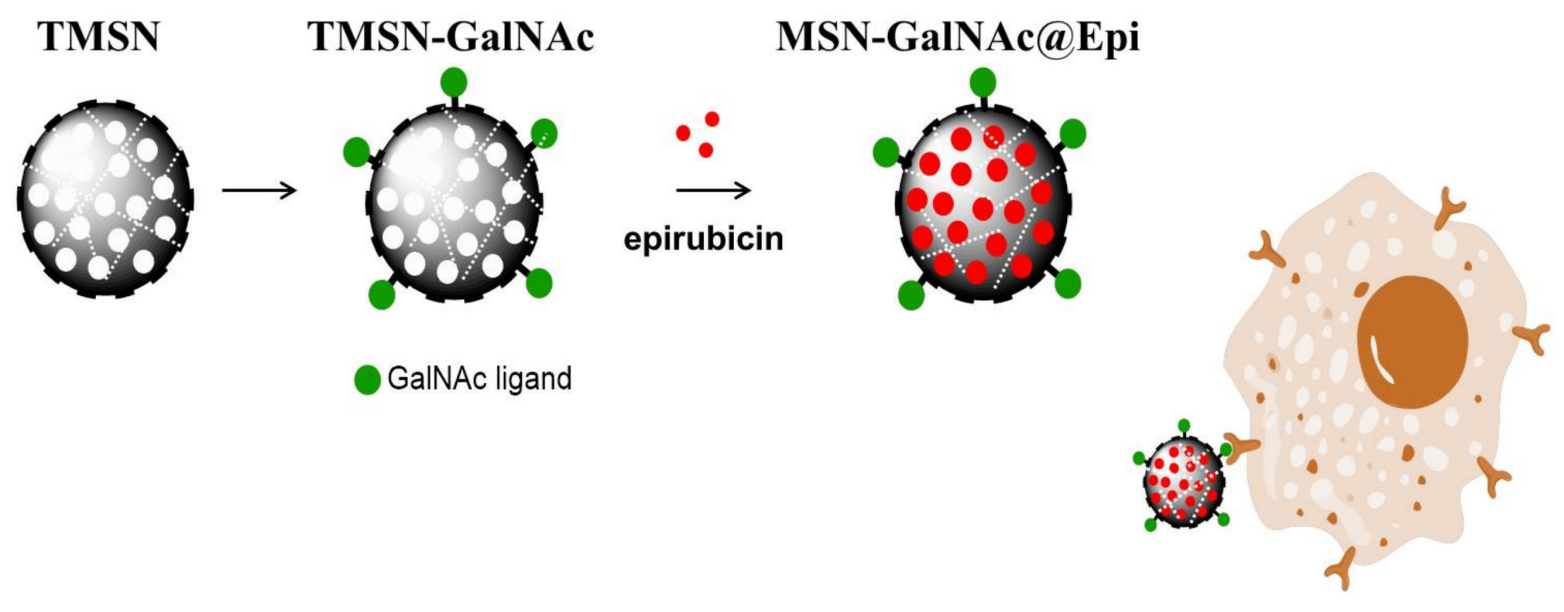
Schematic representation of the developed formulation.

As mentioned before, drug delivery systems can be designed to take advantage of the particular microenvironments of tumors. The HCC microenvironment presents distinctive characteristics such as lower extracellular pH (between 6.0 and 7.0) than normal tissues (7.4) due to its high glycolysis rate and increased steady-state glutathione (GSH) levels, which have been reported to be doubled when compared with normal liver cells [19,20]. Therefore, we envisaged that the development of a targeted and redox-responsive anticancer drug delivery system based on MSNs could be an asset in HCC treatment (Scheme 1). For this purpose, a developed nanosystem formulation consisting of MSNs incorporating tetrasulfide bonds within its structure was developed in order to take advantage of the high GSH levels present both in tumor and intracellular microenvironments. These tetrasulfide-based MSNs (TMSNs) were functionalized with the ligand triantennary GalNAc cluster to confer specificity to HCC cells. These new TMSNs allowed not only a high drug loading capacity of the anticancer drug epirubicin, but also its targeted and controlled release into HCC cells, surpassing critical limitations associated with current clinical treatments (the use of free drugs) and, consequently, contributing to increased therapeutic efficacy.

## 2. Results and Discussion

### 2.1. Synthesis and Characterization of TMSN

**Scheme 2:**
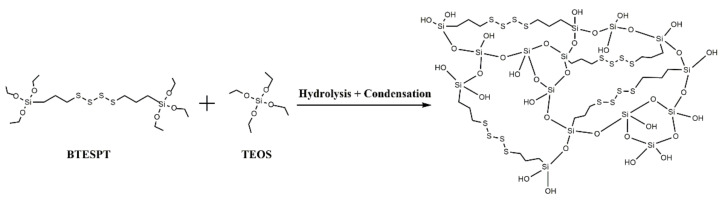
Illustrative scheme of the formation mechanism of a tetrasulfide-based silica matrix.

TMSNs were synthesized through a modified Stöber method using hexadecyltrimethylammoniumbromide (CTAB) as surfactant (acting as pore templating agent) and a mixture of tetraethyl orthosilicate (TEOS) and bis[3-(triethoxysilyl)propyl] tetrasulfide (BTESPT) as silica precursors. BTESPT is used to incorporate tetrasulfide bonds in the silica nanoparticles’ structure (Scheme 2) in order to make them responsive to the redox stimulus.

In order to select the most effective pore induction method, we studied three different approaches for surfactant extraction: sodium carbonate, acidic ethanol, and supercritical drying. As shown in Table 1, depending on the pore induction method used, different physicochemical characteristics were observed for the obtained TMSNs.

The data presented in Table 1 show that Na_2_CO_3_ treatment resulted in partially degraded particles with a heterogeneous and non-porous structure. The visible disruption of the particles could be the main reason for their slightly smaller mean diameter. On the other hand, both the acidic ethanol and the supercritical drying treatments originated round-shaped particles with a porous structure. However, the extreme temperature and pressure conditions of the supercritical drying also led to the breakage of some particles, as shown in TEM image.

Concerning pore diameter, the analysis performed in the sample treated with sodium carbonate was not conclusive, which may be due to the observed deformity and non-porosity of the particles (TEM image). For the two other explored treatments (acidic ethanol and supercritical drying), the data presented in Table 1 show that both approaches resulted in nanoparticles with equal pore diameters of 2.3 nm, which is within the mesoporous range defined by the International Union of Pure and Applied Chemistry (IUPAC) (2–50 nm) [21]. However, TMSNs treated with acidic ethanol revealed a much higher specific surface area and pore volume than those submitted to the supercritical drying. These measurements are in agreement with (and highly reinforce) the observations registered through TEM images since similar pores were observed on both samples, but there was a denser core on TMSNs treated with supercritical drying, which can be correlated with a smaller pore volume and surface area. Moreover, the surface charge (zeta potential) of silica nanoparticles also corroborates the differences between the three treatments. Before treatment, the silica particles displayed a positive ζ potential (+34.1 mV), which is due to the presence of the cationic surfactant (CTAB) used. After the successful removal of CTAB, the surface charge of the particles should be negative due to the presence of the silanol groups (Si-OH) on their surface that become deprotonated in aqueous environment and form –O^−^, leading to negative zeta potentials. From the three different treatments, only the particles treated with acidic ethanol and supercritical drying revealed the expected negative surface charge (−27.5 and −30.7 mV, respectively), demonstrating the successful removal of CTAB. The zeta potential of nanoparticles treated with sodium carbonate did not decrease when compared with non-treated ones, which indicates that the method was not effective. These results suggested that treatment with acidic ethanol was the most effective one. Therefore, this treatment was selected to proceed with the development of the nanosystem.

**Figure 1:**
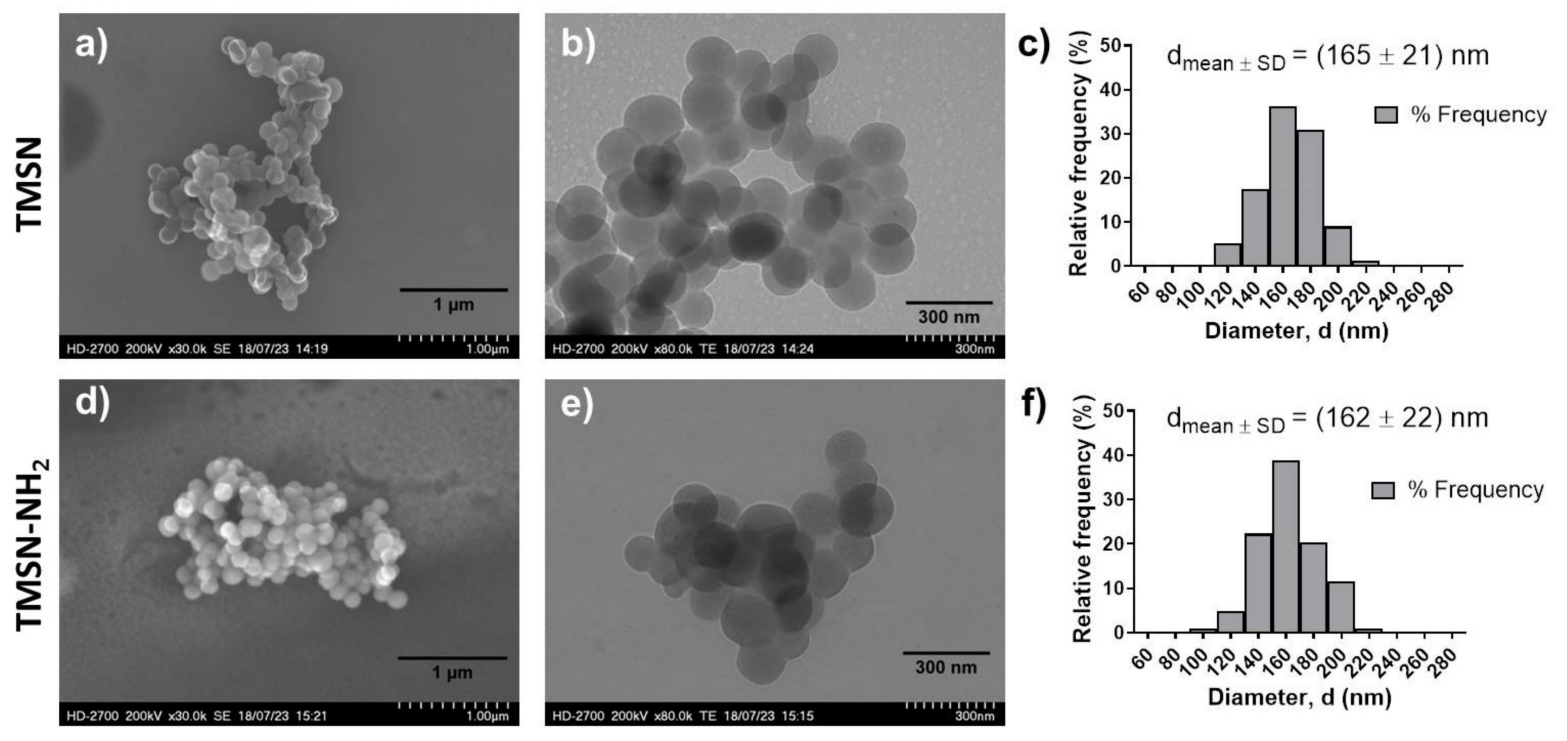
Morphology and size distribution of TMSNs (**a**–**c**) and TMSNs-NH_2_ (**d**–**f**). SEM ((**a**,**d**), scale bar = 1 µm) and TEM ((**b**,**e**), scale bar = 300 nm) images of TMSNs (**a**,**b**) and TMSNs-NH_2_ (**d**,**e**); and histograms showing the particle size distribution measured from the images of TMSNs ((**c**), *n* = 155) and TMSNs-NH_2_ ((**f**), *n* = 103).

After synthesis and surfactant removal procedures, TMSN-OH were modified with APTES in order to provide them amine groups (TMSN-NH_2_) that will posteriorly react with the ligand to make them specific to hepatocellular carcinoma cells. After that, these nanoparticles were physicochemically characterized in order to analyze their morphology, size, and porosity (Figure 1).

As illustrated in Figure 1, the data obtained from SEM and TEM analyses demonstrated that both nanoparticles (TMSNs and TMSN-NH_2_) are round-shaped, present a porous structure, and have a similar and homogeneous size distribution, with average diameters of 165 nm (TMSNs) and 162 nm (TMSN-NH_2_). Moreover, the size distributions show a narrow range of diameters from around 100 to 220 nm in both samples. This similarity in diameters of the particles before and after functionalization is in accordance with results reported in the literature [22]. In actuality, the diameters of the TMSNs depend on the grafted organic group and not on its quantity, and the size of the free APTES molecule, without interactions, is 0.5 nm. Interestingly, when interacting with the particles’ surface, it tends to diminish [23]. To further evaluate the porosity and surface characteristics of the TMSN-NH_2_, nitrogen adsorption–desorption isotherms (Figure S1, Supplementary Materials) were also analyzed. The average pore diameter of 3.6 nm and pore volume of 0.28 cm^2^/g were determined using the Barrett–Joyner–Halenda (BJH) method. These results demonstrated that the developed nanoparticles feature mesopores and high pore volume that could allow a high drug loading capacity. Moreover, the TMSN-NH_2_ have a high specific surface area of 228.0 m^2^/g, obtained from Brunauer–Emmett–Teller (BET) method, which is an interesting characteristic for the surface’s functionalization.

**Figure 2:**
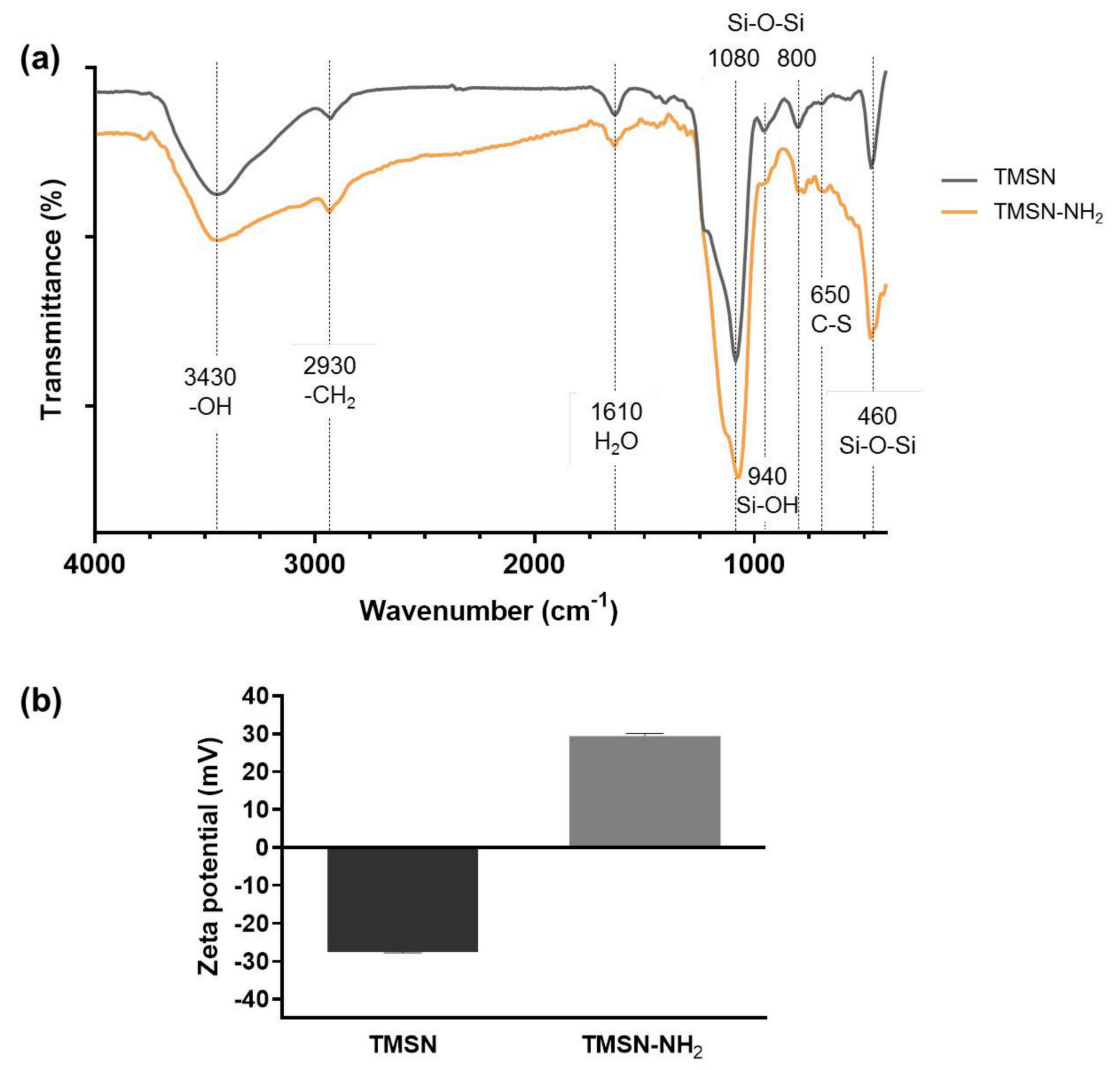
FTIR spectra (**a**) and zeta potential (**b**) of TMSN and TMSN-NH_2_.

To determine the functional groups present on TMSN and TMSN-NH_2_, Fourier-transform IR spectroscopy was performed, and the resulting spectra are shown in Figure 2a, along with the identification of the main characteristic peaks.

As expected, both particles revealed IR peaks typically attributed to silica: Si-O-Si bending at 460 cm^−1^, Si-O-Si stretching vibrations at 800 cm^−1^ and 1080 cm^−1^ with a shoulder around 1200 cm^−1^, and typical Si-OH bending at 946 cm^−1^ [24]. The minor peak at 650 cm^−1^ is attributed to the C-S stretching vibration [25] from the sulfide groups present in BTESPT, whereas the absorption bands at 1610 cm^−1^ have been assigned to water molecules retained by the material [26,27]. The broad peak centered at 3430 cm^−1^ is a common –OH stretching band caused by the presence of the silanol groups [27]. Compared with TMSN, the band on TMSN-NH_2_ is much broader, and it does not return immediately to the baseline, which can be due to the presence of amine groups since the stretches of primary and secondary amines are usually in the region from 3000 to 3400 cm^−1^ [28]. The absorption bands at 2930 cm^−1^ present in the two samples are due to the stretching of CH_2_ [22]. These bands do not appear in typical simple MSN synthesized using only TEOS as the silica precursor [29]; however, they were expected in these TMSN due to the use of BTESPT, as also reported by Moghaddam et al. [30].

Moreover, the functionalization of the TMSN with APTES was also confirmed by the zeta potential (surface charge) measurement of the nanoparticles through electrophoresis. As shown in Figure 2b, a shift from a negative zeta potential value for TMSN (−27.5 mV) to a positive zeta potential value for TMSN-NH_2_ (29.4 mV) was observed. This indicates a successful reaction between TMSN and APTES since the presence of protonated amines on the surface (instead of deprotonated silanol groups) results in positive zeta potential values.

### 2.2. Drug Loading and Drug Release Assays

Epirubicin (Epi) is an anthracycline drug, an analogue of doxorubicin, used in chemotherapy for the treatment of different types of cancer. It interrupts DNA replication—causing cancer cell death—and, when compared to doxorubicin, it presents higher efficacy and fewer side effects, namely cardiotoxicity [31]. TMSN and TMSN-NH_2_ nanoparticles were loaded with epirubicin (TMSN@Epi and TMSN-NH_2_@Epi, respectively), and their loading efficiencies and loading capacities are presented in Table 2.

The Epi loading process was mainly based on physical adsorption of the drug molecules to the nanoparticles. Epirubicin hydrochloride has a pKa of 7.7 in water [32] and a pH of 3 in the injectable solution, thus presenting positive charge. At the solution pH, both the silanol groups on TMSNs and the amino groups on TMSN-NH_2_ are protonated.

However, the surface charge of TMSN-NH_2_ (43.3 ± 1.5 mv) was higher than the one of unfunctionalized TMSN (10.7 ± 2.5 mv), which should result in a higher electrostatic repulsion between TMSN-NH_2_ and Epi, and, consequently, lower drug loading. Nonetheless, as shown in Table 2, the sample that presented the best loading results was, interestingly, TMSN-NH_2_. This enhanced result could be explained by the establishment of hydrogen bonds between the amino groups on the nanoparticles and the drug molecules, which resulted in a stronger interaction than that between silanol groups on TMSNs and Epi, as reported by He et al. [33]. The good drug loading capability of these nanoparticles makes them an attractive platform for drug delivery.

**Figure 3:**
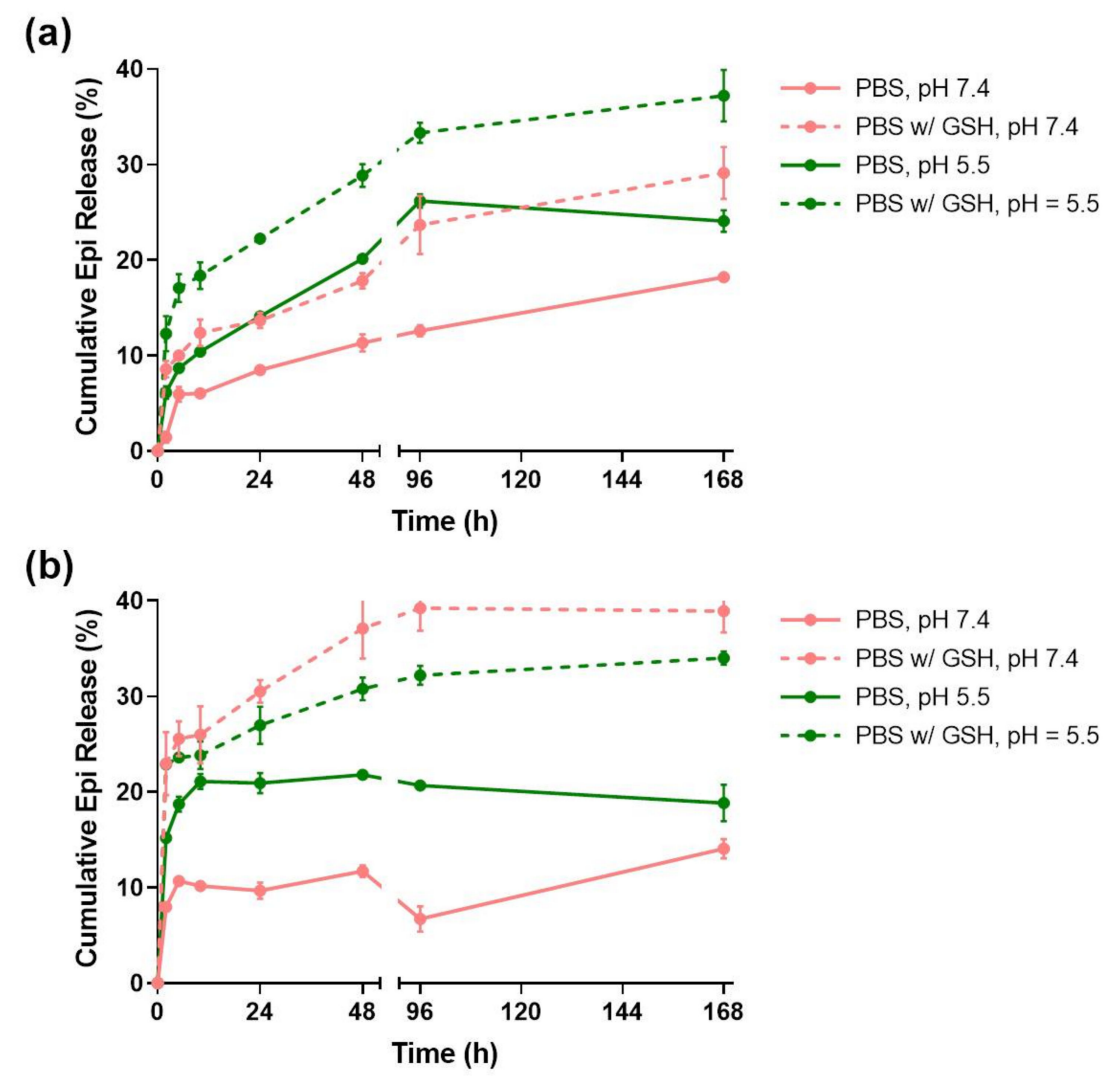
Epirubicin release profiles of TMSN (**a**) and TMSN-NH_2_ (**b**) under relevant biological conditions. Data are expressed as percentage of cumulative epirubicin released and showed as mean ± standard deviation obtained from triplicates.

In order to evaluate the pH and redox responsiveness of the developed drug delivery systems, their release profiles (Figure 3) were studied for 7 days in four different media conditions: PBS at pH = 7.4; PBS with 10 mM GSH at pH = 7.4; PBS at pH = 5.5; and PBS with 10 mM GSH at pH = 5.5.

The TMSNs release profile (Figure 3a) shows that almost 40% of the Epi was released at the end of 168 h in the presence of PBS at pH = 5.5 with 10 mM GSH. However, in the absence of either the acidic pH or GSH conditions, the released drug decreased to around 28%. Additionally, the release in PBS at pH = 7.4 only reached 18%. This data is in agreement with the results obtained by Moghaddam et al. [34] and indicates that unfunctionalized TMSNs are not only redox-responsive, which was expected due to its tetrasulfide bonds, but also pH-responsive. At pH = 7.4, TMSNs are more deprotonated than at pH = 5.5, and thus establish stronger electrostatic interactions with the positively charged Epi molecules.

The release rate of TMSNs-NH_2_ (Figure 3b) was faster than that observed for TMSNs, since at the end of 48 h, the nanoparticles had already reached a plateau on all the different media conditions. Interestingly, this nanosystem seemed to be more redox- than pH-responsive, seeing that 39% and 35% of the drug was released in the presence of GSH at both pH = 7.4 and 5.5, respectively. As in TMSNs, the pH-responsiveness in the absence of GSH arises from the higher protonation of amine groups of the particles at lower pH, leading to enhanced electrostatic repulsions between nanoparticles and Epi [33].

### 2.3. Triantennary GalNAc Functionalized TMSN-NH_2_ as Anticancer Drug Delivery Nanocarriers for Hepatocellular Carcinoma Therapy

**Figure 4:**
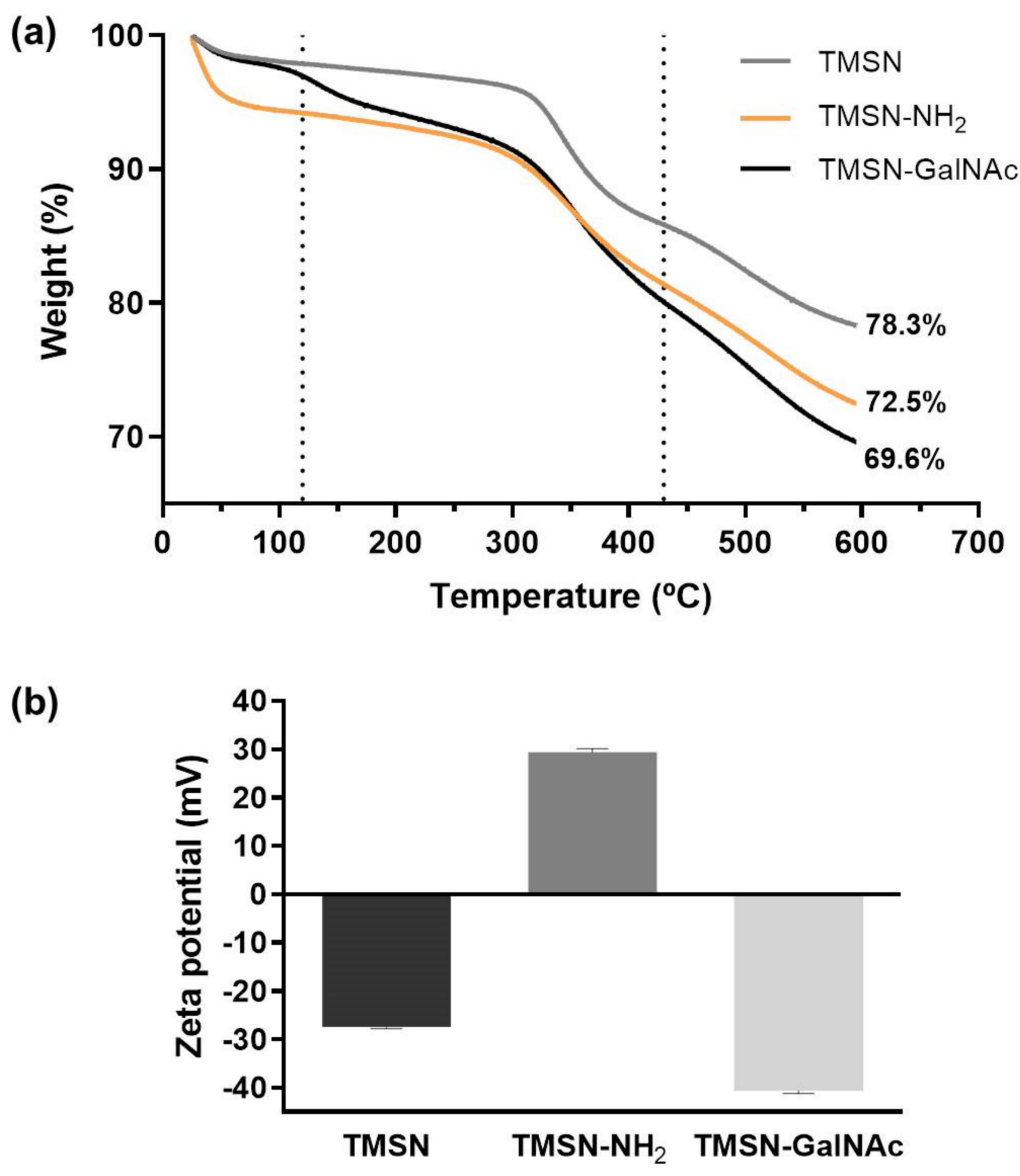
Thermograms (**a**) and zeta potential (**b**) of TMSN, TMSN-NH_2_, and TMSN-GalNAc.

The triantennary *N*-acetylgalactosamine (GalNAc) cluster has been recently explored to target liver cancer cells since it has high affinity for the asialoglycoprotein receptor (ASGP-R), which is overexpressed in this type of cell [5,35]. In order to confer specificity to HCC cells, we selected this cluster as the targeting moiety of our TMSNs, and the conjugation and deprotection reactions were performed according to the procedures reported in the literature [5]. For that, GalNAc pentafluorophenyl ester was firstly attached to the TMSN-NH_2_ and, posteriorly, the O-acetyl groups from the triantennary GalNAc were removed following a procedure already reported in the literature [5]. The content of galactose in TMSN-GalNAc was determined by the resorcinol/sulfuric acid micromethod, and the obtained conjugation ratio was approximately 5%. The TMSN, TMSN-NH_2_, and TMSN-GalNAc were compared through thermogravimetric analysis (TGA), which showed different weight loss profiles while heating the samples from 25 to 600 °C (Figure 4a).

It is noticeable that the total mass loss increased with the functionalization process of the nanoparticles, which suggests an increase in the presence of organic groups on the nanosystems and, at the same time, the success of the functionalization step. In general, there is an initial weight loss until 120 °C, which can be attributed to the removal of ethanol residues or adsorbed water molecules on the surface of particles [22]. In the second temperature range (~120–430 °C), the organic groups of the samples were thermally degraded [22]. TMSN-NH_2_ and TMSN-GalNAc present higher weight loss profiles since they also have a higher organic content due to modification with APTES and trivalent GalNAc clusters, respectively [30,34]. Finally, the mass losses in the third temperature range (~430–600 °C) have been reported to correspond to the removal of water produced by condensation of remaining silanol groups [22].

Moreover, the functionalization of TMSN-NH_2_ nanosystems with the triantennary GalNAc was also confirmed through zeta potential (surface charge) analysis of the nanoparticles (Figure 4b). A shift from a positive zeta potential for TMSN-NH_2_ (29.44 mV) to a negative zeta potential for TMSN-GalNAc (−40.67 mV) was observed, indicating that the reactions between TMSN-NH_2_ and GalNAc successfully occurred, since the presence of the hydroxyl groups of sugar moieties on the surface (instead of protonated amine groups) should result in negative zeta potential values. 

**Figure 5:**
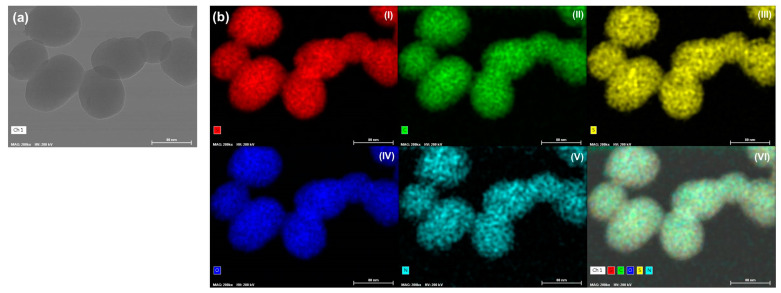
Morphology and elemental mapping analyses for TMSN-NH_2_ functionalized with triantennary GalNAc cluster (scale bar = 80 nm). TEM image (**a**) and EDS maps (**b**): (**I**) silicon (red), (**II**) carbon (green), (**III**) sulfur (yellow), (**IV**) oxygen (dark blue), (**V**) nitrogen (light blue), and (**VI**) overlay image of images (**I**–**V**).

The morphology and elemental composition of the TMSN-GalNAc nanosystems were also observed through STEM-EDS analyses (Figure 5).

TEM image (Figure 5a) of TMSN-GalNAc demonstrated that particles have a uniform and rounded shape with a homogeneous size distribution. In turn, the data obtained in the elemental mapping analyses (Figure 5b) showed the presence of typical elements present in this type of particles: silicon (I), carbon (II), oxygen (IV) and nitrogen (V). Moreover, it was detected the presence of sulfur (III), confirming the incorporation of the silica precursor bis[3-(triethoxysilyl)propyl] tetrasulfide in the silica network of the TMSN, as suggested by the FTIR analysis (Figure 2a) and drug release assay (Figure 3).

### 2.4. Triantennary N-Acetylgalactosamine-Functionalized TMSN-NH_2_ as Drug Delivery Carriers Targeted to Asialoglycoprotein Receptor

**Figure 6:**
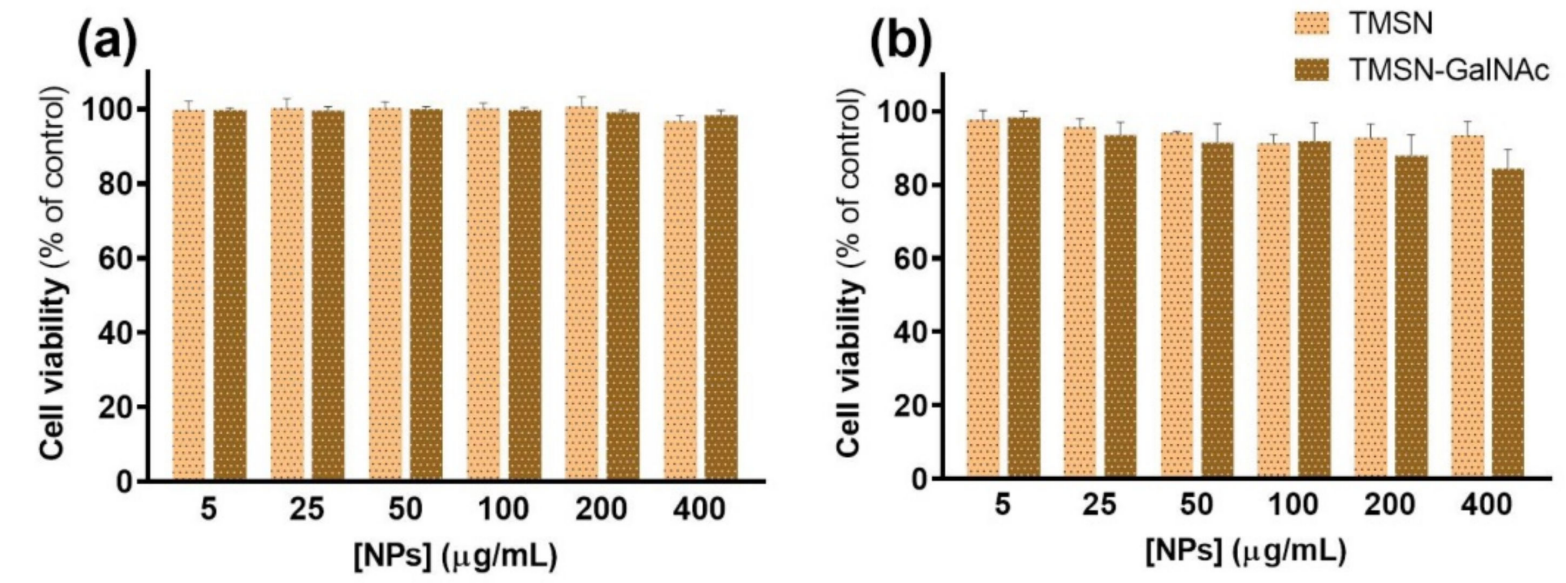
Cell viability of HepG2 cells treated with TMSN and TMSN-GalNAc after 48 h (**a**) and 72 h (**b**) of incubation. Data are presented as a percentage of untreated control cells (mean ± SD), obtained from triplicates, and are representative of at least three independent experiments.

In order to evaluate and compare the potential of the developed TMSNs and TMSN-GalNAc as drug delivery nanocarriers, their cytotoxicity was first analyzed in the absence of drugs. The cellular toxicity promoted by nanoparticles was evaluated after 48 and 72 h of incubation with HepG2 cells, and the obtained results are presented in Figure 6.

The results presented in Figure 6 revealed that both TMSN and TMSN-GalNAc are non-toxic to HepG2 cells after 48 h of incubation, even for high concentrations of nanoparticles (400 µg/mL). After 72 h, cell viability slightly decreased for TMSN-GalNAc, however, the observed levels were always higher than 80%, indicating negligible toxicity and good biocompatibility of the nanosystems, as widely reported in the literature [1,2,34,36].

**Figure 7:**
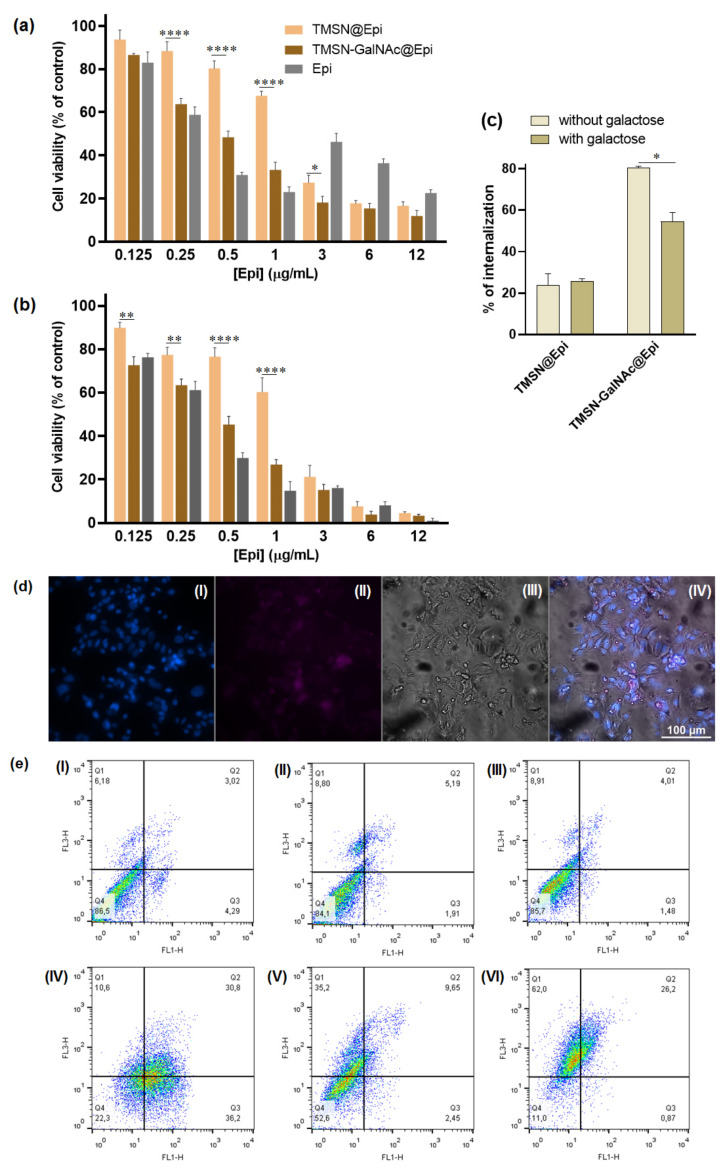
Evaluation and comparison of the impact of TMSN-GalNAc@Epi and TMSN@Epi nanosystems and free epirubicin on cell viability (**a**,**b**), cell internalization (**c**,**d**), and apoptosis (**e**) levels in HepG2 cells. The cell viability was measured 48 h (**a**) and 72 h (**b**) after incubation, and the cell uptake of nanosystems was evaluated in the presence or absence of free galactose (75 mM) after 4 h incubation (**c**). Fluorescence microscopy images (**d**) showing the uptake of the TMSN-GalNAc@Epi: (**I**) cell nuclei stained by Hoechst 33258 dye, (**II**) TMSN-GalNAc@Epi, (**III**) phase contrast image of cells (scale bar = 100 µm). Apoptosis and necrosis levels (**e**) of HepG2 cells were measured 72 h after treatment: (**I**) non-treated, (**II**) TMSN, (**III**) TMSN-GalNAc, (**IV**) free epirubicin, (**V**) TMSN@Epi, and (**VI**) TMSN-GalNAc@Epi. Data were obtained from triplicates and are representative of at least three independent experiments. Asterisks (**** *p* < 0.0001, ** *p* < 0.01, and * *p* < 0.05) denote values that differ significantly.

Thereafter, TMSN-GalNAc nanoparticles were evaluated as a targeted anticancer drug delivery system to HCC cells. For this purpose, TMSN-GalNAc particles were loaded with epirubicin (TMSN-GalNAc@Epi), exhibiting a loading efficiency and loading capacity of 20.89% and 8.36%, respectively. Moreover, the Fourier-transform infrared spectra for TMSN and TMSN-GalNAc loaded with epirubicin also confirmed the loading ability of both TMSN and TMSN-GalNAc (Figure S2, Supplementary Materials) by the presence of characteristic peaks of epirubicin, the C=O stretching frequency (1680 cm^−1^), and the stretching vibration of the CH_2_ group (1385 cm^−1^), as reported in the literature [36]. The therapeutic efficacy mediated by this formulation, TMSN-GalNAc@Epi, was compared with the one mediated by unmodified TMSNs loaded with Epi (TMSN@Epi) in HepG2 cells (Figure 7).

In order to correlate the amount of Epi with the therapeutic potential of the TMSN-GalNAc@Epi and TMSN@Epi nanosystems, after 48 h and 72 h of incubation with HCC cells, different concentrations of Epi-loaded nanoparticles were evaluated. The toxicity profile of Epi-loaded nanosystems at both 48 h and 72 h revealed an expected linear decrease in cell viability with the increase in Epi concentration from 0.125 to 12 µg/mL. Generally, TMSN-GalNAc@Epi induced higher cytotoxicity than non-targeted TMSN@Epi, denoting the influence of the presence of the triantennary GalNAc cluster at the surface of the nanosystem. This promoted higher cell binding and internalization of the nanosystems (Figure 7c,d), consequently promoting a higher drug delivery efficiency and thus an improved cell death effect. This is particularly visible for epirubicin concentrations of 0.5 and 1 µg/mL. This toxicity profile was also observed in the flow cytometry apoptosis/necrosis levels evaluation (Figure 7e), achieving 22.5%, 55.6%, and 11% of viable cells after treatment with free Epi, TMSN@Epi, and TMSN-GalNAc@Epi, respectively.

To clarify if the association of the GalNAc ligand to nanosystems promoted the potentiation of their cell internalization by the specific interaction of GalNAc with ASGP-R, a competitive inhibition study using galactose as a specific ligand for ASGP-R was performed. For this purpose, HepG2 cells were incubated with 75 mM of free galactose 30 min before nanosystems incubation, as reported in the literature [37]. The data presented in Figure 7c show that the presence of free galactose promoted a significant decrease in the cellular uptake of TMSN-GalNAc@Epi nanosystems but not in those prepared without the GalNAc ligand (TMSN@Epi). Moreover, the results also reveal the significant difference found in the cell internalization ability of targeted and non-targeted nanosystems, indicating that the binding and internalization of these TMSN-GalNAc nanocarriers to HCC cells are promoted by the specific interaction of the GalNAc ligand with the ASGP-R.

## 3. Conclusions

In this work, a redox-responsive silica-based nanocarrier functionalized with the triantennary *N*-acetylgalactosamine (GalNAc) cluster was developed to exhibit high affinity for the asialoglycoprotein receptor, which is overexpressed in liver cancer cells. This new TMSN-GalNAc nanosystem showed great biocompatibility, high drug loading capacity, and efficient and specific drug delivery in hepatocellular carcinoma cells, inducing a high HCC cell death effect. Overall, the results showed that the newly developed nanosystem could be a promising approach for future clinical applications in liver disease treatment since it has the potential to surpass the main constraints associated with current chemotherapy strategies. Moreover, the new nanosystem can be easily adapted for other drug therapeutics targeting liver cells.

## 4. Materials and Methods

### 4.1. Materials

Tetraethyl orthosilicate (TEOS, ≥99.0%), (3-aminopropyl)triethoxysilane (APTES, ≥99.0%), bis[3-(triethoxysilyl)propyl] tetrasulfide (BTESPT, ≥90.0%), Dulbecco’s modified Eagle’s medium-high glucose (DMEM-HG), fetal bovine serum (FBS), L-gluthathione reduced (GSH, ≥98.0%), resazurin sodium salt, sodium carbonate (Na_2_CO_3_, ≥99.0%), sodium hydroxide (NaOH, ≥97.0%), and sodium chloride (NaCl, ≥98.0%) were received from Sigma-Aldrich. Hexadecyltrimethylammoniumbromide (CTAB, ≥98.0%) was acquired from Merck. Dimethyl sulfoxide (DMSO, ≥99.7%), hydrochloric acid (HCl, 37%), and absolute ethanol were received from Thermo Fisher Scientific. All materials were used as received and without further purification. Ultrapure water was obtained using a Milli-Q^®^ water system (Millipore, Burlington, MA, USA). Epirubicin hydrocloride solution for injection (2 mg/mL, Teva Pharmaceutical Industries, Ltd., Petah Tivka, Israel) (Epi) was kindly provided by IPO-Centro (Coimbra, Portugal) and triantennary *N*-acetylgalactosamine (GalNAc) cluster was synthesized and kindly offered by Ionis Pharmaceuticals (Carlsbad, CA, USA).

### 4.2. Methods

#### 4.2.1. Synthesis of Mesoporous Tetrasulfide-Based Silica Nanoparticles (TMSN-OH)

Mesoporous tetrasulfide-based silica nanoparticles were synthesized according to a previously reported procedure in the literature [30]. Briefly, 110 mL of ultrapure water, 10 mL of absolute ethanol, 220 mg of CTAB, and 900 µL of an aqueous solution of NaOH (2M) were mixed in a 250 mL round-bottomed flask at 80 °C and under reflux at a stirring rate of 500 rpm for 30 min. Then, the stirring rate was increased to 1400 rpm and a mixture of TEOS and BTESPT with the molar ratio of 6.6:1 was added dropwise to the solution. The reaction was left under stirring for 6 h. After synthesis, nanoparticles were precipitated and washed three times with ethanol by centrifugation at 18,000 rpm and 4 °C for 10 min (Avanti J-26 XPI Centrifuge (Beckman Coulter, CA, USA)) and maintained in alcohol. The nanoparticles were suspended in acidic ethanol (1 mL of HCl 36.5% in 30 mL of ethanol 100%) and kept under reflux at 80 °C and 1500 rpm for 6 h. Then, they were washed three times with water and freeze-dried.

#### 4.2.2. Functionalization of the TMSN-NH_2_ with Triantennary *N*-Acetyl Galactosamine (TMSN-GalNAc)

TMSNs-OH were firstly functionalized through a post-synthetic grafting of APTES. Typically, 50 mg of TMSNs-OH were sonicated in 25 mL of water and placed under magnetic stirring. APTES was added dropwise to the suspension and the reaction was allowed to stir at 500 rpm for 24 h. The nanoparticles (TMSN-NH_2_) were collected by centrifugation, washed three times with water, and freeze-dried and stored at room temperature for further use.

The attachment of the triantennary GalNAc to the TMSN-NH_2_ was adapted from a procedure reported in the literature [5]. Briefly, 15 mg of TMSN-NH_2_ was sonicated in 2 mL of DMSO and 1 mL of a solution of GalNAc at 40 mM was added to the suspension. The reaction was performed at room temperature at 500 rpm for 4 h. After that period of time, 10 mL of ammonium solution at 25% was added to the suspension for ligand deprotection (removal of O-acetyl groups). After 3 h of reaction, the nanoparticles were washed twice with DMSO and once with water, freeze-dried, and stored at 4 °C until use.

### 4.3. Physicochemical Characterization of the Developed Nanosystem

#### 4.3.1. Zeta Potential Analysis

The zeta potential (surface charge) of the nanoparticles was obtained through laser Doppler electrophoresis. These analyses were performed in a Zetasizer Nano ZS equipment using a disposable folded capillary cell DTS1070 (ZetaSizer Nanoseries, Malvern Instruments, Malvern, UK). All the samples were suspended in ultrapure water at a concentration of 100 μg/mL and sonicated for 10 min immediately before analysis.

#### 4.3.2. Transmission Electron Microscopy

Transmission electron microscopy (TEM) was used to explore the size and morphology of the nanoparticles. They were suspended in water and sonicated for 10 min previous to the analysis. After that, a small sample of the suspension was deposited on formvar/carbon-coated grids (Electron Microscopy Sciences, Hatfield, PA, USA), and after 1 min, the samples were dried by touch with filter paper to remove the excess of water. The observations were carried out on a Tecnai G2 20 S-Twin electron microscope (FEI) at 200 kV. For size analysis, TEM images were examined using ImageJ to measure the nanoparticle diameters, and their distributions were presented as histograms, along with the mean ± SD.

#### 4.3.3. Scanning Transmission Electron Microscopy and Energy-Dispersive X-ray Spectroscopy (STEM-EDS)

The size and morphology of the samples were also evaluated by scanning transmission electron microscopy (STEM), which allowed us to obtain both TEM and SEM (scanning electron microscopy) images. Additionally, STEM was coupled to an energy-dispersive X-ray spectroscopy (EDS) for the elemental analysis of samples. The nanoparticles were suspended in ethanol and sonicated for 2 min, after which a small sample was dropped on Formvar-carbon coated grids (Electron Microscopy Sciences, Hatfield, PA, USA) and let to dry overnight. All the measurements were done on a Hitachi HD-2700, type B STEM, operated at 200 kV and the EDS was a Fisons EA1108 CHNS-O Element Analyzer, operated with the furnace at 900 °C and with a helium flow of 120 mL/min.

#### 4.3.4. Nitrogen Adsorption–Desorption Isotherms

N_2_ adsorption–desorption isotherm analyses were conducted on a Micromeritics ASAP 2000 in order to measure the surface area, pore size, and pore volume of the nanoparticles. The Brunauer–Emmett–Teller (BET) specific surface area was calculated using the adsorption data at P/P0 = 0.05–0.32. Pore diameter and volume were acquired from a desorption branch using the Barrett–Joyner–Halenda (BJH) method.

#### 4.3.5. Fourier-Transform Infrared (FTIR) Spectroscopy

Fourier-Transform Infrared Spectroscopy (FTIR) was performed in order to determine the chemical structure of the nanoparticles and its functional groups. The measurements were performed using the KBr pellet method, which explores potassium bromide’s (KBr) property of becoming plastic when subjected to pressure and involves forming a thin pellet that is transparent in the IR region. The preparation of the samples was performed by mixing 0.2–0.3 mg of the sample with 78.5–80 mg of KBr in a mortar and grinding it in order to obtain a homogeneous mixture. The samples were then dried overnight at 40 °C to ensure that any remaining water was eliminated and pressed in a pellet-forming die. Before performing the sample measurements, a background reading was performed using a pellet of KBr only. All the measurements were performed using a Jasco FT/IR 4200 spectrometer with 256 scans and a resolution of 4 cm^−1^ in the range between 400 and 4000 cm^−1^.

#### 4.3.6. Resorcinol/Sulfuric Acid Micromethod

The amount of galactose in TMSN-GalNAc was determined by the resorcinol/sulfuric acid micromethod, adapting a procedure reported in the literature [38]. Briefly, 20 µL of suspension of TMSN or TMSN-GalNAc in water (75 µg/µL) was mixed with 20 µL of resorcinol aqueous solution (6 µg/µL) and 100 µL of sulfuric acid (75%), followed by incubation for 30 min at 90 °C and then another 30 min at room temperature in the dark. The absorbance of the compounds formed in the chromogenic reaction was measured at 492 nm through the SpectraMax Plus 384 spectrophotometer. The grafting ratio of galactose in the MSN was calculated using a galactose standard curve.

#### 4.3.7. Thermogravimetric Analysis (TGA)

Thermogravimetric analyses (TGA) were performed in order to estimate the degree of functionalization of the nanoparticles both with APTES and with the GalNAc cluster. This technique is a destructive analysis in which the weight loss of the sample is monitored while it is heated along the desired temperature range. All the tests were conducted on a TGA Q500 (TA Instruments, New Castle, DE, USA). During the measurements, the samples were heated from room temperature to 600 °C at a heating rate of 10 °C/min under a nitrogen atmosphere.

#### 4.3.8. Drug Loading and Release

The loading of the anticancer drug Epirubicin (Epi) into the nanoparticles was achieved through an adapted procedure [3,24]. Typically, 5 mg of nanoparticles was suspended in 1 mL of Epi (2 mg/mL), and the obtained suspension was stirred at room temperature in the dark for 24 h. The resulting suspension was centrifuged at 4000 rpm for 10 min (Rotofix 32 A Centrifuge (Hettich, Tuttlingen, Germany)) and washed with ultrapure water twice to remove the unloaded drug. All the supernatants of the three centrifugations were collected and stored at 4 °C, protected from light. The washed NPs were freeze dried under vacuum overnight and stored at 4 °C until further use. The quantification of the loaded Epi was performed using UV–Vis absorption spectroscopy. According to the usual procedure, 50 μL of the supernatants was added to 950 μL of ultrapure water in order to achieve a dilution factor of 20, to avoid equipment saturation. The amount of loaded Epi was calculated by subtracting the amount of unloaded drug collected in the supernatants to the amount of initial feeding drug and then the loading efficiency (LE) and loading capacity (LC) were calculated according to the equations below:

$$LE\left(wt\%\right)=\left(\frac{weight\;of\;loadeddrug}{weight\;of\;feedingdrug}\right)\times 100\;LC\left(wt\%\right)=\left(\frac{weight\;of\;loaded\;drug}{weight\;of\;nanoparticles}\right)\times 100$$

For the release studies, Epi-loaded TMSNs were dispersed in phosphate-buffered saline (PBS) (0.2 μg/mL) with or without 10 mM of GSH at different pH values 7.4 or 5.5. The suspensions of nanoparticles were sonicated for 1 min in order to obtain homogeneous suspensions and then equally distributed between 7 Eppendorfs with 1 mL each. All the Eppendorfs were kept under constant shaking (160 rpm) at 37 °C, in a shaking incubator, and one selected Eppendorf of each group was taken out at specific time points (2, 5, 10, 24, 48, 96, and 168 h) and centrifuged at 16,000 rpm for 10 min in an Eppendorf^®^ Centrifuge 5415D. After that, the supernatant was collected in a new Eppendorf and centrifuged again for another 10 min. The amount of Epi in the supernatant was then measured through UV–Vis absorption. 

Regarding the UV–Vis absorption spectroscopy, triplicates of 100 μL of each sample and each standard (calibration curve) were transferred to a 96-well microplate with a clear bottom, and the absorbance was measured in a spectrophotometer SpectraMax Plus 384 (Molecular Devices, San Jose, CA, USA) at 480 nm.

#### 4.3.9. Cytotoxicity Assays

The cytotoxicity of unloaded and Epi-loaded TMSNs and TMSN-GalNAc nanoparticles was evaluated in HepG2 cell line, which is derived from the liver tissue of a 15-year-old Caucasian American male with well-differentiated HCC. The cells were maintained at 37 °C, under 5% CO_2_ in DMEM-HG, supplemented with 10% (*v*/*v*) heat-inactivated FBS, penicillin (100 U/mL) and streptomycin (100 μg/mL). For the cytotoxicity evaluation, cells were seeded onto 48-well plates at a density of 80,000 cells/well and incubated to grow for 24 h. After that, the culture media were removed and different conditions were added to the wells: for the unloaded TMSNs and TMSN-GalNAc, fresh media contained various nanoparticle concentrations ranging from 0 to 800 μg/mL; for Epi-loaded TMSNs and TMSN-GalNAc, samples were prepared based on Epi concentrations ranging from 0 to 12 μg/mL. Wells with media and without nanoparticles were used as control. The cells were then incubated for another 48 and 72 h, after which cell viability was evaluated by the Alamar Blue Assay. Briefly, the cells were incubated with 0.3 mL of 10% (*v*/*v*) resazurin (0.1 mg/mL stock solution) in DMEM medium at 37 °C. After 1.5 h of incubation, 180 μL of the supernatant from each well was transferred to a 96-well plate and their absorbances were measured at 570 and 600 nm (SpectraMax Plus 384 spectrophotometer (Molecular Devices, San Jose, CA, USA)). Cell viability was presented as a percentage (mean ± SD) of untreated control cells and calculated according to the formula (A_570_–A_600_) of treated cells × 100/(A_570_–A_600_) of control cells.

Cytotoxicity was also evaluated by flow cytometry using the probes FITC-annexin V (Immunostep) and propidium iodide (PI) (Sigma, St. Louis, MO, USA). After 72 h of incubation with epirubicin-loaded nanosystems or free drug at a concentration of 0.5 µg/mL, HepG2 cells were trypsinized, washed with PBS, and resuspended in 100 µL of binding buffer (2.5 mM CaCl_2_, 10 mM HEPES (pH 7.4) and 140 mM NaCl) to which 2 µL of FITC-annexin V (0.05 mg/mL) and 1 µL of PI (0.05 mg/mL) were added. Samples were incubated for 5 min in the dark and, after that, analyzed in a FACSCalibur flow cytometer (Becton Dickinson, Franklin Lakes, NJ, USA). FITC fluorescence was evaluated in the FL-1 channel, PI was evaluated in the FL-3 and 20,000 events were collected. The data were analyzed using CellQuest software.

#### 4.3.10. Competition Assay

For the competition assay of TMSN-GalNAc with galactose, HepG2 cells seeded 24 h before were preincubated with or without galactose (75 mM) for 30 min, and then TMSN-GalNAc@Epi and TMSN@Epi nanoparticles were added to the culture media and incubated for 4 h. Then, cells were washed with PBS and harvested by trypsinization. Afterwards, cells were centrifuged at 1000 rpm for 5 min, resuspended in 0.3 mL of PBS, and examined using the forward scatter (FSC)/side scatter (SSC) and fluorescence parameters in a FACSCalibur flow cytometer (Becton Dickinson, Franklin Lakes, NJ, USA), where 20,000 events were collected for each sample. The data were analyzed using CellQuest^TM^ software.

#### 4.3.11. Statistical Analysis

Data are presented as mean result ± standard deviation (SD) and analyzed using GraphPad Prism (version 9, GraphPad Software Inc., San Diego, CA, USA). Statistical significance of differences between data was evaluated by two-way ANOVA using Sidak’s multiple comparison test. A *p*-value < 0.05 was considered as statistically significant.

## Figures and Tables

**Table 1:** Physicochemical characterization by transmission electron microscopy (TEM image—scale bar = 50 nm), Brunauer–Emmett–Teller/Barrett–Joyner–Halenda and zeta potential analyses of TMSNs obtained after treatment with sodium carbonate, acidic ethanol, and supercritical drying.

Pore Induction Method	TEM Image	Average Pore Diameter (nm)	Specific Surface Area (m^2^/g)	Pore Volume (cm^3^/g)	Zeta Potential (mV)
Sodium carbonate	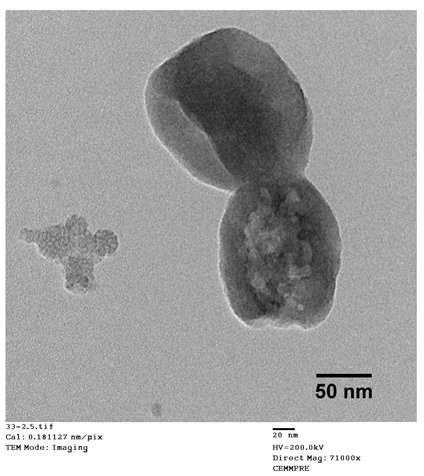	-	-	-	+34.6
Acidic ethanol	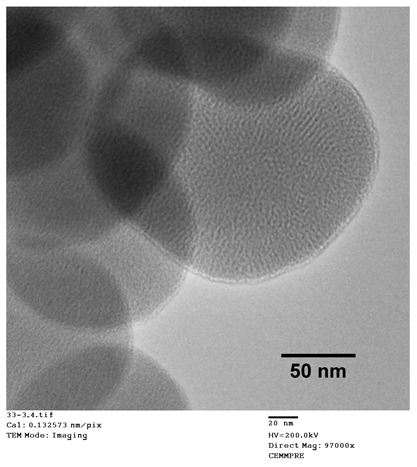	2.3	745.3	0.57	−27.5
Supercritical drying	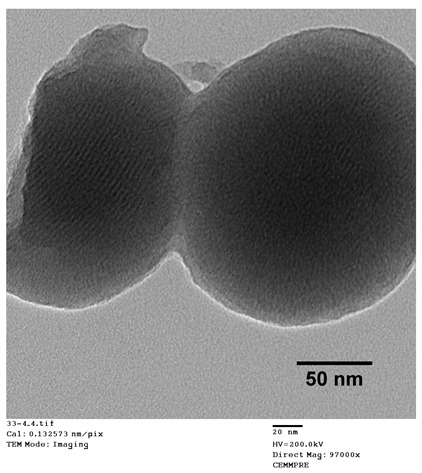	2.3	420.5	0.31	−30.7

**Table 2:** Drug loading efficiency and loading capacity of TMSN@Epi and TMSN-NH_2_@Epi (data are presented as means ± SD).

	Loading Efficiency (wt%)	Loading Capacity (wt %)
TMSN@Epi	17.2 ± 1.6	6.9 ± 0.7
TMSN-NH_2_@Epi	23.3 ± 7.5	9.3 ± 3.0

## Supplementary Materials

The following supporting information can be downloaded at: www.mdpi.com/article/10.3390/ijms23116243/s1.
