# Supplementary material for: Triantennary GalNAc-Functionalized Multi-Responsive Mesoporous Silica Nanoparticles for Drug Delivery Targeted at Asialoglycoprotein Receptor
Source: Int J Mol Sci. 2022 Jun 2;23(11):6243. doi: 10.3390/ijms23116243 (PMC9181004; doi:10.3390/ijms23116243)
Supplement: Supplementary file 1 [file ijms-23-06243-s001.zip › ijms-1582082-supplementary.pdf]

Supplementary Materials

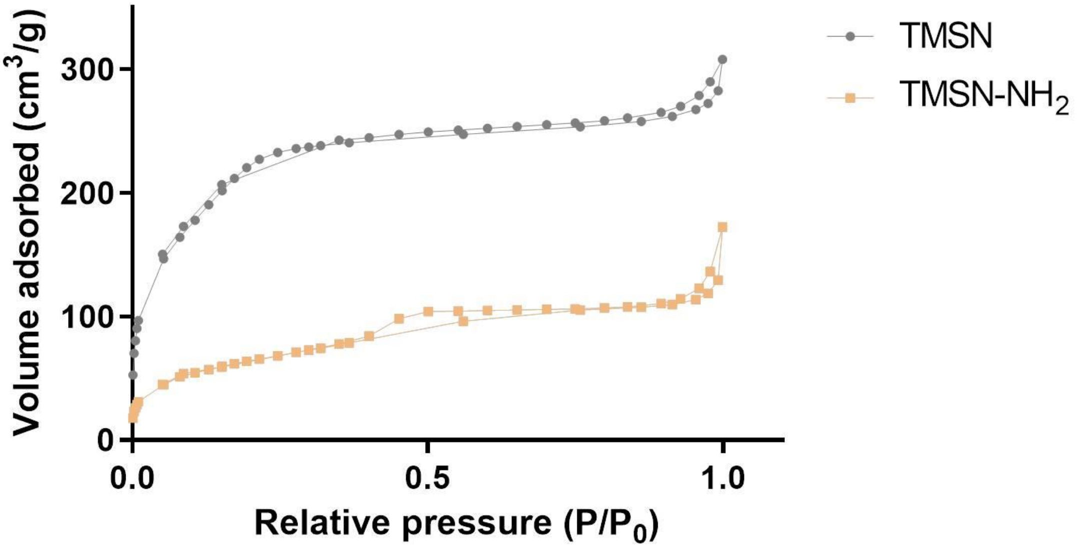

Figure S1. Nitrogen adsorption-desorption isotherms of TMSN and TSMN-NH_2.

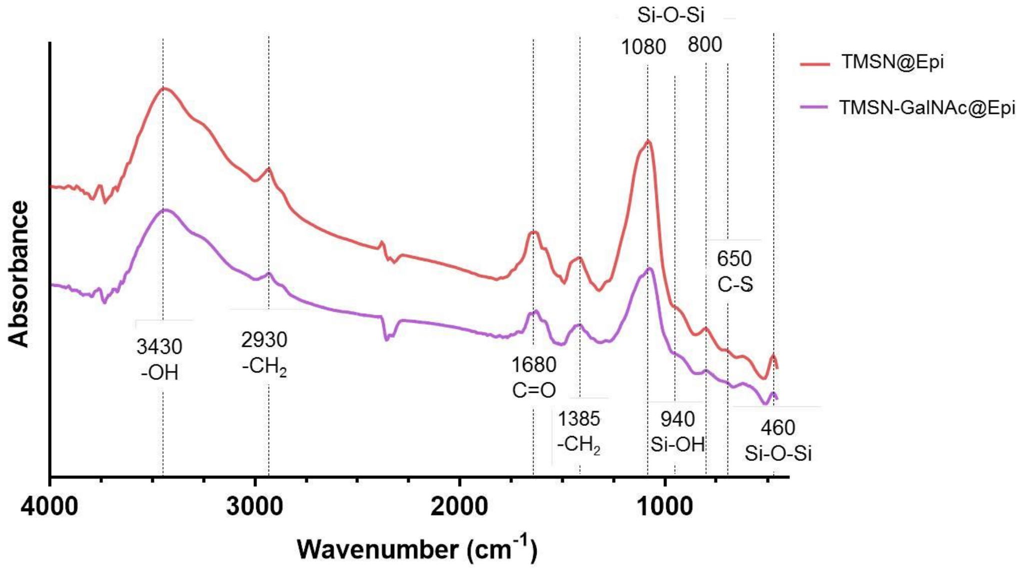

Figure S2. FTIR spectra of TMSN and TMSN-GalNAc loaded with epirubicin, represented by TMSN @Epi and TMSN-GalNAc@Epi, respectively.
